# Functional morphology of the primary olfactory centers in the brain of the hermit crab *Coenobita clypeatus* (Anomala, Coenobitidae)

**DOI:** 10.1007/s00441-020-03199-5

**Published:** 2020-04-02

**Authors:** Marta A. Polanska, Tina Kirchhoff, Heinrich Dircksen, Bill S. Hansson, Steffen Harzsch

**Affiliations:** 1 Department of Animal Physiology, Institute of Zoology, Faculty of Biology, University of Warsaw, 1 Miecznikowa Street, 02-096 Warsaw, Poland; 2 Zoological Institute and Museum, Department of Cytology and Evolutionary Biology, University of Greifswald, Soldmannstrasse 23, 17498 Greifswald, Germany; 3 Department of Zoology, Stockholm University, Svante Arrhenius väg 18B, SE-10691 Stockholm, Sweden; 4 Max-Planck-Institute for Chemical Ecology, Department of Evolutionary Neuroethology, Hans-Knöll-Straße 8, 07745 Jena, Germany

**Keywords:** *Coenobita*, Terrestrial hermit crab, Olfactory system

## Abstract

Terrestrial hermit crabs of the genus *Coenobita* display strong behavioral responses to volatile odors and are attracted by chemical cues of various potential food sources. Several aspects of their sense of aerial olfaction have been explored in recent years including behavioral aspects and structure of their peripheral and central olfactory pathway. Here, we use classical histological methods and immunohistochemistry against the neuropeptides orcokinin and allatostatin as well as synaptic proteins and serotonin to provide insights into the functional organization of their primary olfactory centers in the brain, the paired olfactory lobes. Our results show that orcokinin is present in the axons of olfactory sensory neurons, which target the olfactory lobe. Orcokinin is also present in a population of local olfactory interneurons, which may relay lateral inhibition across the array of olfactory glomeruli within the lobes. Extensive lateral connections of the glomeruli were also visualized using the histological silver impregnation method according to Holmes-Blest. This technique also revealed the structural organization of the output pathway of the olfactory system, the olfactory projection neurons, the axons of which target the lateral protocerebrum. Within the lobes, the course of their axons seems to be reorganized in an axon-sorting zone before they exit the system. Together with previous results, we combine our findings into a model on the functional organization of the olfactory system in these animals.

## Introduction

Multiple times during their evolutionary radiation, representatives of several malacostracan taxa have independently invaded the terrestrial habitat. We are interested in the question how new selection pressures related to the evolutionary conquest of land have reshaped sensory systems of terrestrial Crustacea, specifically their olfactory system (review Hansson et al. 2011). Our studies on aerial olfaction in crustaceans have focused on the taxon Coenobitidae (Anomala, Paguroidea), which comprises two genera that display a fully terrestrial life style (McLaughlin et al. 2007), 15 species of shell-carrying land hermit crabs (the genus *Coenobita*) and the robber or coconut crab *Birgus latro* (genus *Birgus*). This genus is a relatively “young” group in evolutionary terms, as evidenced from its fossil records (Luque 2017). An evolutionary adaptation to terrestrial conditions must require physiological adaptations related to gas exchange, maintenance of ion balance, or osmoregulation. Such adaptations must also concern the sensory systems, which, in the case of olfaction, require complex detection of airborne cues in form of hydrophobic volatile substances in the gas phase. In fact, behavioral studies have suggested that members of the Coenibitidae effectively detect volatile odors (Rittschof and Sutherland 1986; Vannini and Ferretti 1997; Stensmyr et al. 2005). These omnivorous crabs are attracted by chemical cues emitted by as different sources as seawater, well water, and distilled water (Vannini and Ferretti 1997), crushed conspecifics or snails (Thacker 1996), fruits, seeds, flowers (Rittschof and Sutherland 1986; Thacker 1996, 1998), and even horse feces and human urine (Rittschof and Sutherland 1986).

**Fig. 1:**
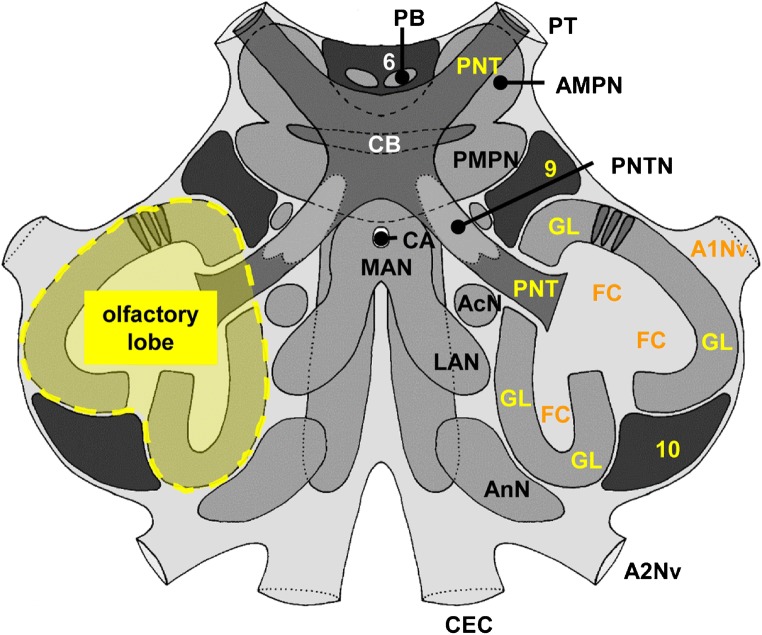
*Coenobita clypeatus*, scheme of the median brain (modified from Harzsch and Hansson 2008). The left olfactory lobe is highlighted in yellow. Neuronal elements of the central olfactory pathway are identified with yellow and orange letters. A1Nv antenna 1 nerve, A2Nv antenna 2 nerve, AcN accessory lobe, AMPN anterior median protocerebral neuropil, AnN antenna 2 neuropil, CA cerebral artery, CB central body, CEC circumesophageal connectives, FC fibrous core, GL glomerular layer, LAN lateral antenna 1 neuropil, MAN median antenna 1 neuropil, PB protocerebral bridge, PMPN posterior median protocerebral neuropil, PNT projection neuron tract, PNTN neuropil of the projection neuron tract, PT protocerebral tract, numbers 6, 9, 10: cell clusters (6), (9), and (10)

The basic structure of the crustacean central olfactory pathway has been thoroughly analyzed in large decapods, namely, crayfish, clawed lobsters, and spiny lobsters (reviews e.g. Mellon and Alones 1993; Sandeman and Mellon 2002; Schachtner et al. 2005; Schmidt 2007; Schmidt and Mellon 2011; Derby and Weissburg 2014; Sandeman et al. 2014; Derby et al. 2016; Harzsch and Krieger 2018). They use the most anterior pair of head appendages, their deutocerebral antennae, as the main chemosensory organ for distance olfaction. The axons of olfactory sensory neurons (OSNs) associated with aesthetasc sensilla on the antennae project into the brain’s primary olfactory centers, the bilaterally paired olfactory lobes (Fig. 1). There, the afferents establish synaptic contacts with two classes of olfactory interneurons, the local olfactory interneurons and the olfactory projection neurons within specialized neuropil compartments, the olfactory glomeruli which are spherical or cone-shaped synaptic fields, which act as the fundamental processing units of the olfactory system (reviews: Sandeman and Mellon 2002, Schachtner et al. 2005, Schmidt and Mellon 2011, Derby and Weissburg 2014, Sandeman et al. 2014, Schmidt 2016). Complex stimuli are thought to be encoded using a “population” or “across-neuron pattern code” in which differences in the activity between glomeruli are essential (Schmidt 2007; Derby and Weissburg 2014). The glomeruli typically are regionalized into functional compartments along their long axis, and in representatives of the crayfish (Blaustein et al. 1988; Sandeman and Luff 1973), clawed lobsters (Langworthy et al. 1997), spiny lobsters (Blaustein et al. 1988; Schmidt and Ache 1992, 1996, 1997; Wachowiak and Ache 1997; Wachowiak et al. 1997), and hermit crabs (Harzsch and Hansson 2008; Krieger et al. 2010, 2012), an outer cap, a subcap, and a base region can be distinguished. This compartmentalization, which is also mirrored in the glomerular neurochemistry (Langworthy et al. 1997; Schmidt and Ache 1997; Polanska et al. 2012), suggests that complex local processing occurs at the level of single glomeruli. Another type of olfactory interneurons, the projection neurons, represent the output pathway of the system, and their axons assemble in the projection neuron tract to target specific protocerebral areas.

In members of the Coenobitidae, the aesthetasc sensilla were described as short and blunt (Ghiradella et al. 1968), a morphology that was suggested to minimize water evaporation while maintaining the ability to detect volatile odorants in gaseous phase (Stensmyr et al. 2005). These animals display active chemoreception behaviors, and the kinematics of antennal flicking and its role in odorant capture have been dealt with elsewhere (Waldrop and Koehl 2016; Waldrop et al. 2016). Because the aesthetasc surface is lacking any visible pores, the question how volatile molecules penetrate the cuticle is still unclear (Tuchina et al. 2014). Antennal glands associated with the aesthetascs fields in *C. clypeatus* generate mucus that may be involved in antimicrobial defense and in providing moisture crucial for the absorption of odorant molecules (Tuchina et al. 2014). Electroantennography (EAG) recordings of *B. latro* (Stensmyr et al. 2005) and *C. clypeatus* demonstrated the capacity of excised terminal antennule segments to detect volatile chemicals, and in *C. clypeatus*, dose-dependent responses to water-soluble carboxylic acids and mono- and diamines but also to volatile aldehydes were reported (Krång et al. 2012). Sequencing antennal transcriptomes identified 29 putative ionotropic receptors (IRs) in *C. clypeatus* (Groh et al. 2014), and their expression within the olfactory sensory neurons was documented (Groh-Lunow et al. 2015).

Using immunohistochemistry, we have studied the general organization of the brain and of the central olfactory pathway in both *C. clypeatus* (Harzsch and Hansson 2008) and *B. latro* (Krieger et al. 2010). Furthermore, the innervation pattern of the primary olfactory center by the OSN afferents was explored (Tuchina et al. 2015), and the neurochemical diversity of local olfactory interneurons has been analyzed earlier by immunohistochemistry against RFamide-like and allatostatin-like neuropeptides (Polanska et al. 2012). The organization of the protocerebral hemiellipsoid bodies, the higher integrative centers, which are targeted by the olfactory projection neurons, was studied by immunohistochemistry (Wolff et al. 2012) and on an ultrastructural level (Brown and Wolff 2012). Collectively, these studies indicate that Coenobitidae have highly complex central olfactory systems. The current study sets out to expand our knowledge of the functional morphology of the primary olfactory center in *C. clypeatus* by using classical neuroanatomical techniques. Furthermore, here we continue to explore the neurochemical complexity of the system by using antisera against RFamide-related peptides, allatostatin-like peptides, and serotonin. In addition, we newly employed an antiserum against orcokinins, a highly conserved family of neuropeptides identified in many crustacean and insect species (Bungart et al. 1994; Hofer et al. 2005; Hofer and Homberg 2006a, b; Tanaka 2016). Our results provide new insights into the termination of the afferents in the lobe, aspects of lateral inhibition, and structural organization of the output pathway of the system.

## Material and methods

### Embedding in paraffin and Azan stain

Adult specimens of *Coenobita clypeatus* (Herbst, 1791; Anomala Coenobitidae) were obtained and reared as described in Harzsch and Hansson (2008) and Polanska et al. (2012). Before dissection, the animals were anesthetized for at least 1 h on ice. Then, the animals were decapitated and their heads placed immediately in Dubosq-Brasil fixative (with Gregory’s modification (Gregory 1980), a mixture of 120 ml absolute alcohol, 30 ml distilled water, 1 g picric acid, 60 ml 40% formaldehyde, and 15 ml glacial acetic acid that was artificially aged at 60 °C for several days. The specimens were kept in the fixative for 48 h at room temperature. The brains were then dissected from the cephalothorax and dehydrated in an ascending ethanol series (80, 90, 96, 100, 100% EtOH, each step 20 min at RT) and in several changes of 100% xylene, until the brains sank to the bottom of the vial. Dehydrated tissues were first infiltrated for 1 h at 60° in a 1:1 mixture of xylene and paraplast followed by three 1-h changes of paraplast (melting point 58 °C; Sigma) in an oven at 60 °C, embedded in blocks, cut into 10-μm sections with a rotary microtome (Microm HM 360, Fisher Scientific), and mounted on gelatin-coated glass slides. Following paraffin removal and rehydration in descending ethanol series, the sections were used for a morphological analysis. The serial sections were stained with Azan-novum in the modification by Geidies according to the standard protocols described by Lang (2006) and Mulisch and Welsch (2010).

### Silver impregnation

For a detailed structural study of *C. clypeatus* brain, we applied the classical histological silver impregnation method according to Holmes-Blest (Humason 1979; Blest and Davie 1980) with the modifications described in Sandeman et al. (1993). Following paraffin removal and rehydratation, the sections were impregnated in 20% silver nitrate bath (1.18 mol/l) in constant darkness for 48 h at room temperature. Next, slides were rinsed for 5 min in distilled H_2_O and incubated in a freshly prepared bath solution containing 27.5 ml 0.2 M Boric acid Roth, (6943.2), 22.5 ml 0.05 M Borax (Roth, 8643.1), 20 ml 1% silver nitrate (Roth, 7908.2), and 10 ml pyridine, filled-up with distilled H_2_O to 250 ml (pH 8.4), for 24 h at 37 °C. To develop the color, slides were immersed into a developer solution containing 3 g hydroquinone and 15 g sodium sulfite in 300 ml of distilled H_2_O heated to 60 °C for approx. 5 min. Subsequently, slides were washed for 5 min in running tap water and rinsed in distilled H_2_O. As a next step, slides were toned for 3–5 min in 2% gold chloride solution (Gold(III) chloride trihydrate; Sigma-Aldrich, G4022-1G) until sections turned colorless. As a reduction step, after a brief bath in distilled H_2_O, slides were kept in two to three changes of 2% oxalic acid solution until the sections turned blue. Thereafter, specimens were washed in distilled H_2_O and fixed for 5 min in 5% sodium thiosulfate. Finally, slides were thoroughly washed in running tap water and distilled water and then dehydrated in an ascending ethanol series (30%, 50%, 60%, 70%, 80%, 90%, 96%, 2 × 100%), cleared twice in 100% xylene and coverslipped with Entellan medium (VWR 1.07960.0500).

### Immunohistochemistry

Immunohistochemistry was carried out as described previously for *C. clypeatus* (Harzsch and Hansson 2008; Polanska et al. 2012). The brains were isolated from animals anesthetized by cooling on ice for at least 30 min. Brains were dissected in phosphate—buffered saline (0.1 M PBS, pH 7.4) and fixed overnight in 4% paraformaldehyde (PFA) in PBS at 4 °C. After fixation, tissues were washed in several changes of PBS for at least 4 h at room temperature, embedded in low-gelling agarose (Cat. A9414; Sigma-Aldrich Chemie GmbH, Munich, Germany), and cut on a vibratome (Zeiss Hyrax V-50) into 80-μm sections. As free-floating sections, these were permeabilized in PBSTx (0.3% Triton x-100 in 0.1 M PBS, pH 7.4) for 1 h at RT and incubated in the primary antisera diluted in PBSTx overnight at 4 °C. The antisera we used were monoclonal anti-SYNORF1 synapsin antibody (DSHB, 3C11; from mouse; 1:10 dilution; RRID: AB_2313867), polyclonal anti-A-allatostatin antiserum (A-type Dip-allatostatin I; Jena Bioscience, abd-062; from rabbit; 1:1000 dilution; RRID: AB_2314318), polyclonal anti-serotonin (5-HT, Immunostar, Cat. No 20080, from rabbit, igG; 1:1000 dilution; RRID: AB_572263), and polyclonal anti-orcokinin (from rabbit, 1:200 Bungart et al. 1994; Dircksen et al. 2000). After incubation in the primary antisera, the sections were washed in several changes of PBS for 1 h and afterwards incubated in the secondary antibodies (anti IgGs) conjugated to Alexa Fluor 488 (1:1000; Alexa Fluor 488 goat anti-rabbit IgG Antibody, Invitrogen, Thermo Fisher Scientific; Waltham, MA, USA; RRID: AB_10374301) and Cy3 (1:1000; Cy3-conjugated AffiniPure Goat Anti-Mouse IgG Antibody, Jackson ImmunoResearch Laboratories Inc.; West Grove, PA, USA; RRID: AB_2338000.) overnight at room temperature. Subsequently, a 1-h incubation in Hoechst 33342 dye (Invitrogen) diluted 1:10,000 in PBS served as a nuclear counterstain. Some sections were processed with a histochemical counter stain, a high-affinity probe for actin, by adding phallotoxins conjugated to Alexa Fluor 546 (Molecular Probes; concentration 200 units/ml) to the secondary antibody in a dilution 1:50.The sections were finally washed in several changes of PBT for 2 h and mounted in Mowiol 4–88 (Cat. 0713.2; Carl Roth). Our analysis is based on more than five successfully processed brains per marker, and the labeling pattern was consistent between these specimens. We are carried out controls for all markers in which the primary antibody was replaced by PBS. In these controls, labeling was abolished completely (data not shown).

### Antibody specificity

#### Synapsin

The monoclonal anti-SYNORF1 synapsin antibody (DSHB Hybridoma Product 3C11; anti SYNORF1 as deposited to the DSHB by E. Buchner, University Hospital Würzburg, Germany; supernatant) was raised against a *Drosophila melanogaster* GST-synapsin fusion protein and recognizes at least four synapsin isoforms (70, 74, 80, and 143 kDa) in western blots of *D. melanogaster* head homogenates (Klagges et al. 1996). Sullivan and co-workers (2007) mention a single band at approx. 75 kDa in a western blot analysis of crayfish brain homogenate. Harzsch and Hansson (2008) conducted a western blot analysis comparing brain tissue of *D. melanogaster* and the hermit crab *Coenobita clypeatus* (Anomura, Coenobitidae). The SYNORF1 serum provided identical results for both species, and it stained one strong band between 80 and 90 kDa and a second weaker band slightly above 148 kDa, suggesting that the epitope that SYNORF1 recognizes is strongly conserved between *D. melanogaster* and *C. clypeatus* (see Harzsch and Hansson 2008). Similar to the fruit fly, the antibody consistently labels brain structures in other major subgroups of the malacostracan crustaceans (e.g., Beltz et al. 2003; Harzsch et al. 2002, 1999, 1998; Krieger et al. 2012) in a pattern that is consistent with the assumption that this antibody labels synaptic neuropils in crustaceans.

#### Serotonin

The antiserum against serotonin (ImmunoStar Incorporated; Cat. No. 20080, Lot No. 541016) is a polyclonal rabbit antiserum raised against serotonin coupled to bovine serum albumin (BSA) with paraformaldehyde. The antiserum was quality control–tested by the manufacturer using standard immunohistochemical methods. According to the manufacturer, staining with the antiserum was completely eliminated by pretreatment of the diluted antibody with 25 μg of serotonin coupled to BSA per ml of the diluted antibody. We repeated this control with the serotonin-BSA conjugate that was used for generation of the antiserum as provided by ImmunoStar (Cat. No. 20081, Lot No. 750256; 50 μg of lyophilized serotonin creatinine sulfate coupled to BSA with paraformaldehyde). Preadsorption of the antibody in working dilution with the serotonin-BSA conjugate at a final conjugate concentration of 10 μg/ml at 4 °C for 24 h completely blocked all immunolabeling. We performed an additional control and preadsorbed the diluted antiserum with 10 mg/ml BSA for 4 h at room temperature. This preadsorption did not affect the staining, thus, providing evidence that the antiserum does not recognize the carrier molecule alone. The manufacturer also examined the cross reactivity of the antiserum. According to the data sheet, with 5 μg, 10 μg, and 25 μg amounts, the following substances did not react with the antiserum diluted to 1:20,000 using the horse radish peroxidase (HRP) labeling method: 5-hydroxytryptophan, 5-hydroxyindole-3-acetic acid, and dopamine. The antiserum was previously used to label brain structures in *C. clypeatus* (Harzsch and Hansson 2008).

#### Allatostatin

The A-type allatostatins represent a large family of neuropeptides that were first identified from the cockroach *Diploptera punctata*; they additionally share the C-terminal motif -YXFGLamide (Christie et al. 2010; Nässel and Homberg 2006; Stay et al. 1995; Stay and Tobe 2007). In the shore crab *Carcinus maenas* (Brachyura), almost 20 native A-type allatostatin-like peptides were identified from extracts of the thoracic ganglia (Duve et al. 1997). Shortly afterwards, various other A-type allatostatin-like peptides were isolated from the Spiny Cheek crayfish *Orconectes limosus* (Astacida; Dircksen et al. 1999). Meanwhile, A-type allatostatin (AST-A)-like peptides have been discovered in a wide range of malacostracan crustaceans, including Brachyura (e.g. Huybrechts et al. 2003), Astacida (e.g. Cape et al. 2008), the prawns *Penaeus monodon* (Duve et al. 2002), *Macrobrachium rosenbergii* (Yin et al. 2006), and also the shrimp *Penaeus vannamei* (Ma et al. 2010; Meth et al. 2017). Yasuda-Kamatani and Yasuda (2006) have shown that contain more than 25 closely related ASTA-like peptides occur on the same crayfish *Procambarus clarkii* allatostatin-like peptide precursor and could thus be co-released. Christie (2016) predicted a total of 29 peptides with the C-terminal motif, -YXFGLamide, in the latest bioinformatic analysis on the peptidome of the shore crab. The polyclonal rabbit allatostatin antiserum used in the present study was raised against the *Diploptera punctata* A-type Dip-allatostatin I,APSGAQRLYGFGLamide, coupled to bovine thyroglobulin using glutaraldehyde (Vitzthum et al. 1996). It has previously been used to localize A-type allatostatin-like peptides in crustacean and insect nervous systems (e.g., Kreissl et al. 2010; Polanska et al. 2012). In the following, the term “allatostatin-like immunoreactivity” is used to indicate that the antibody most likely binds to various related peptides within this peptide family.

#### Orcokinin

The first orcokinin was discovered almost three decades ago in extracts (Stangier et al. 1992) and later in neurons of the abdominal nerve cord of the crayfish *Orconectus limosus* (Astacida; Dircksen et al. 2000). Yasuda-Kamatani and Yasuda (2000, 2006) identified two different orcokinin gene products in the crayfish *P. clarkii* showing that the cloned mRNA precursors gave rise to multiple copies of the first discovered and name-giving Asn^13^-orcokinin but also to single copies of another four isoforms with modified C-terminal or internal amino acids; all orcokinin-isoforms were also identified biochemically in this study. Today, orcokinins are known to represent a highly conserved family of neuropeptides identified in many crustacean and insect species (Bungart et al. 1994, Hofer et al. 2005, Hofer and Homberg 2006a, b). In crustaceans, these neuropeptides are widely distributed in the nervous system and display strong myotropic and neuromodulatory activities (Bungart et al. 1994, 1995; Dircksen et al. 2000; Li et al. 2002). In most of the hitherto investigated insects, orcokinins are one amino acid longer (14 amino acids) and may play different roles than in crustaceans. Experiments on the cockroach *Leucophaea maderae* proved that orcokinins are engaged in the regulation of locomotor activity controlled by circadian clock neurons (Hofer and Homberg 2006b). In the silk moth *Bombyx mori*, orcokinins were described as neuronal protothoracicotropic factors regulating the biosynthesis of ecdysteroids (Yamanaka et al. 2011). Recently, by use of transcriptome shotgun assembly (TSA) datasets, orcokinin-encoding transcripts were predicted in spiders (Christie and Chi 2015), and, by immunocytochemistry, orcokinin-immunoreactive neurons were identified in ticks (Roller et al. 2015). We used a rabbit anti-Asn^13^-orcokinin (Asn^13^-OK) antiserum (Bungart et al. 1994) that was raised against a glutaraldehyde-conjugate of bovine thyroglobulin and Asn13-OK. Most likely because of the very conserved N-terminal sequence NFDEIDR- in most orcokinins discovered to date, the Asn^13^-OK-antiserum showed almost full cross-reaction with Val^13^-orcokinin (Dircksen et al. 2000) and likely all other C-terminally modified crustacean and even identified insect (cockroach *Rhyparobia* = *Leucophaea maderae*) orcokinins (Hofer et al. 2005) showing this C-terminal sequence. This sequence is, however, missing or almost entirely changed in some still so-called insect orcokinins (Sterkel et al. 2012; Chen et al. 2015; Wulff et al. 2017).

### Imaging

The brain tissues processed for immunofluorescence were viewed with a Leica TCS SP5II confocal laser-scanning microscope equipped with Diode- and Argon-lasers and operated by the Leica “Application Suite Advanced Fluorescence” software package (LASAF) (Leica Microsystems, Wetzlar, Germany). Digital images were processed with Adobe Photoshop CS4 or ImageJ. Only global picture enhancement features (brightness and contrast) were used. The brain tissues processed for histology were viewed with a Nikon Eclipse 90i upright microscope and bright-field optics (Nikon, Amstelveen, Netherlands).

### Nomenclature

The neuroanatomical nomenclature used in this manuscript for neuropils, clusters of cell bodies and tracts is based on Sandeman et al. (1993) and Richter et al. (2010) with some modifications adopted from Krieger et al. (2012) and Loesel et al. (2013). The olfactory globular tract is named the projection neuron tract (PNT) according to Loesel et al. (2013). Cell clusters are referred by their given numbers in parentheses according to the nomenclature from Sandeman et al. (1992).

## Results

### Overall structure of the primary chemosensory centres

**Fig. 2:**
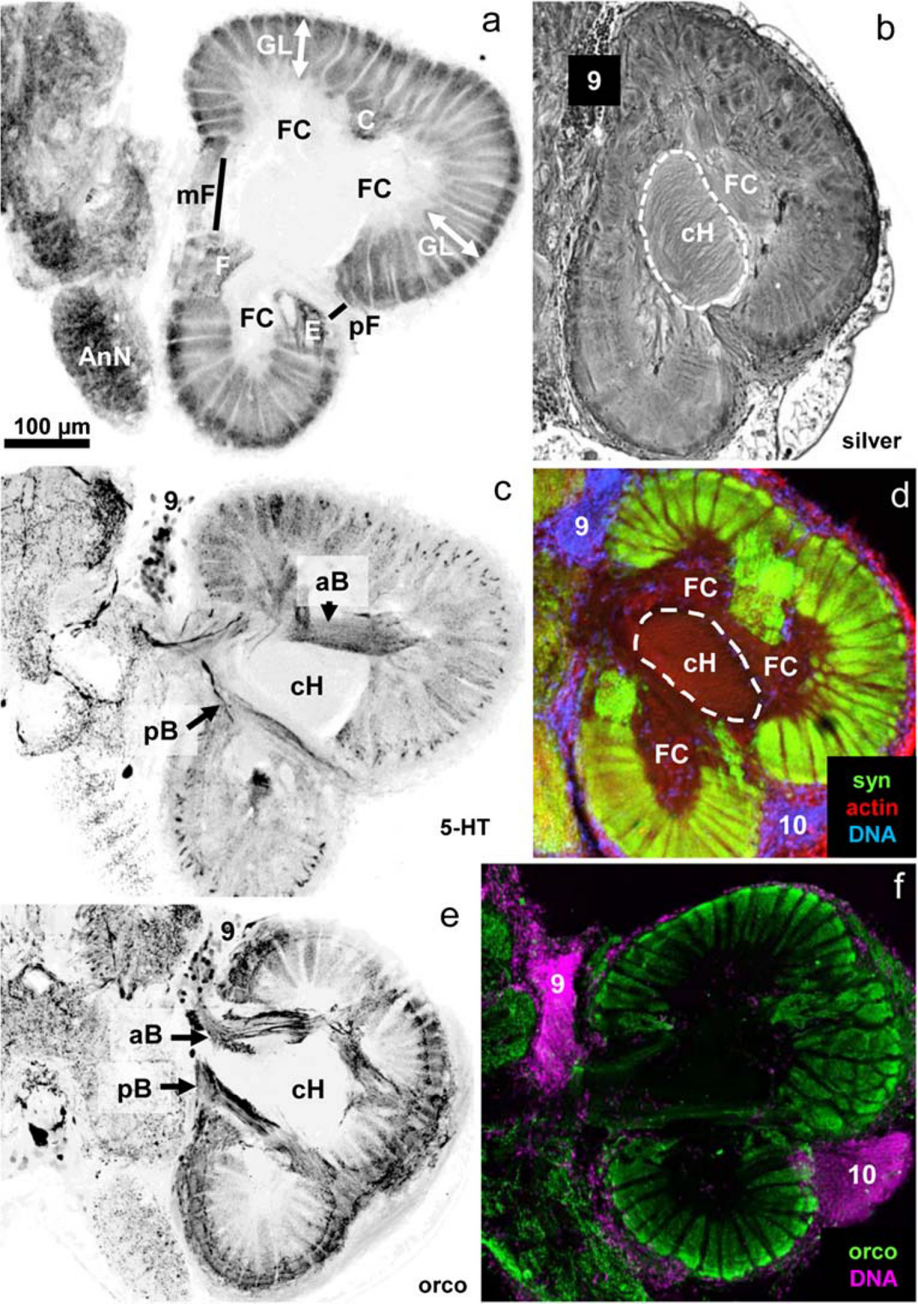
Right olfactory lobe visualized with different histological techniques (the midline is to the left, and anterior to the top). **a** Synapsin-immunoreactivity; **b** silver impregnation; **c** serotonin-immunoreactivity; **d** synapsin-immunoreactivity (green), actin (red), DNA (blue); **e, f** orcokinin-immunoreactivity (green), DNA (magenta). aB anterior bundle, cH central projection neuron hub, AnN antenna 2 neuropil, FC fibrous core, GL glomerular layer, mF medial foramen, pB posterior bundle, pF lateral foramen, numbers 9, 10: cell cluster (9), (10), letters C/E/F: non-glomerular neuropils C/E/F; The scale bar in **a** applies for all figures in the panel

The primary deutocerebral chemosensory centres, the olfactory lobes, are spherical, bilaterally paired lobes (Fig. 1). In the following, we will restrict our description to the center of only one side of the brain, typically the right side. Immunohistochemistry against presynaptic vesicle proteins to label synapse-dense areas reveals an array of wedge-shaped synaptic units, which are radially arranged around a non-synaptic fibrous core (FC; Fig. 2a, b). These synaptic units traditionally are called olfactory glomeruli despite their elongated shape because they take on a spherical form in phylogenetically more ancestral crustacean taxa so that spherical olfactory glomeruli characterize the crustacean ground pattern (Harzsch and Krieger 2018). We will call the entire array of glomeruli the “glomerular layer” (Gl) in the following (double arrows in Fig. 2a). There are also a few patches of non-glomerular synaptic neuropil, which are identified with letters “C,” “E,” and “F” in Fig. 2a according to the nomenclature established in Harzsch and Hansson (2008). The fibrous core in the center of the lobe can be visualized using the silver impregnation method (Fig. 2b) and also a fluorescent probe against actin (red in Fig. 2d). In these preparations, an additional array of parallel fibers can be recognized in the center of the fibrous core, which we will term “central projection neuron hub” (cH) in accordance with the terminology established by Ito et al. (2014), and which we will describe in more detail below. The glomerular layer is interrupted by a median (mF) and a posterior (pF) foramen (Fig. 2a) across which the neurites of olfactory interneurons take their course into and out of the lobe. The somata of these neurons are assembled in cell clusters (9), (10) (Fig. 2d, f), and (11) (not shown). Immunohistochemistry for serotonin (Fig. 2c) and orcokinin-like neuropeptides (Fig. 2e) labels the somata of local olfactory interneurons within cluster (9), the neurites of which enter the lobe across the median foramen. After the passage across the foramen, these neurites assemble in an anterior (aB) and a posterior bundle (pB) that extend around the central hub to innervate the glomerular layer (Fig. 2c, e). Immunohistochemistry against serotonin and orcokinin-like neuropeptides (Fig. 2c, e, and f) shows that these substances are not evenly distributed across the proximal to distal axis of individual glomeruli but instead indicates the presence of local morphological compartments. These are known as “cap,” “subcap,” and “base” structures (Harzsch and Hansson 2008; Polanska et al. 2012) and will be highlighted in more detail below.

### Orcokinin immunoreactivity is present in the afferents from the olfactory sensory neurons

**Fig. 3:**
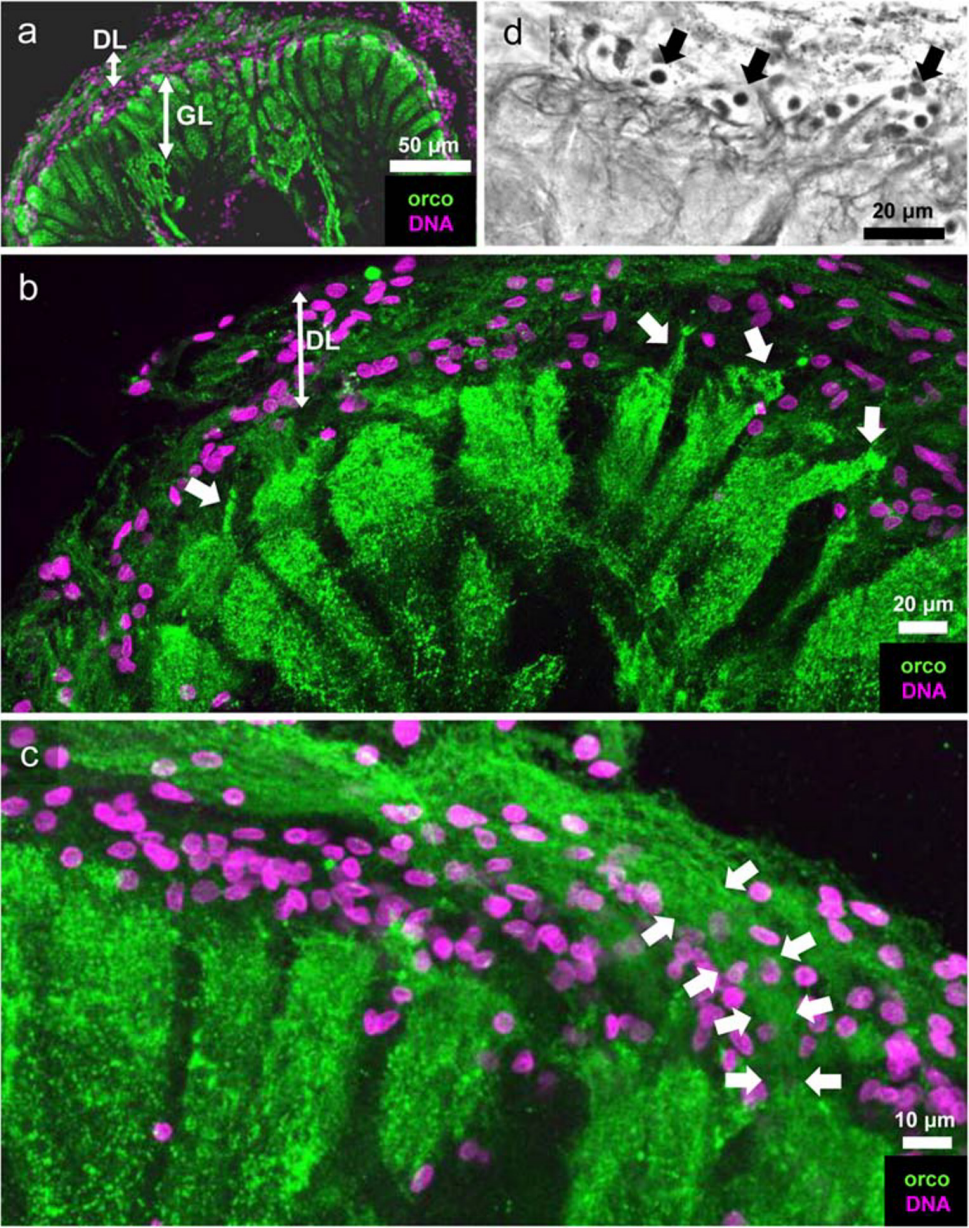
**a** Anterior part of the olfactory lobe, orcokinin-immunoreactivity (green) and cell nuclei (magenta) in the distal layer and glomerular layer (the midline is to the left, and anterior to the top). **b** Higher magnification of the interface between distal layer and glomerular layer. Note the abundant cell nuclei (magenta) in the distal layer. The glomeruli are strongly orcokinin-immunoreactive (green) and irregular extensions (arrows) seem to protrude from the cap region of the glomeruli into the distal layer. **c** Higher magnification of the distal layer showing numerous cell nuclei (magenta) and a plexus of orcokinin-immunoreactive fibers (green) from which bundles (arrows)—presumably the axons of olfactory sensory neurons—dive into the glomerular cap region. **d** Silver impregnation, interface of distal layer and glomerular layer. Arrows point to glial cell nuclei. DL distal layer, GL glomerular layer

In large decapod crustaceans (Sandeman and Luff 1973; Schmidt and Ache 1992; Mellon and Alones 1993) including *C. clypeatus* (Tuchina et al. 2015), the axons of the olfactory sensory neurons form a specific branch of the antenna 1 nerve and spread out laterally over the outer surface of the primary chemosensory center from where they dive vertically into the olfactory glomeruli to innervate their distal part. In *C. clypeatus*, immunohistochemistry for orcokinin strongly labels the synaptic volumes of all glomeruli and also a layer of fibrous material (“distal layer”, DL; Fig. 3a, b) with a parallel, tangential texture that distally covers the lobe. Numerous cell nuclei, presumably belonging to glia cells, are embedded within the distal layer. Some of the glomeruli display irregular, strongly immunolabeled extensions, which protrude into (or from) the fibrous layer (arrows in Fig. 3b). In some preparations, bundles of immunolabeled fibers appear to extend from the distal layer into the distal part of the olfactory glomeruli (arrows in Fig. 3c). These may represent bundles of afferents. Cell nuclei were also visible within the distal layer in silver impregnations probably representing glia cells (arrows in Fig. 3d). These preparations also showed irregularly arranged fibrous material at the interface between the distal layer and the glomerular layer. However, it was impossible to determine from our preparations if these fibers represent afferent axons or originate from other cells, e.g., the glia cells themselves.

### Orcokinin immunoreactivity is also present in local olfactory interneurons

**Fig. 4:**
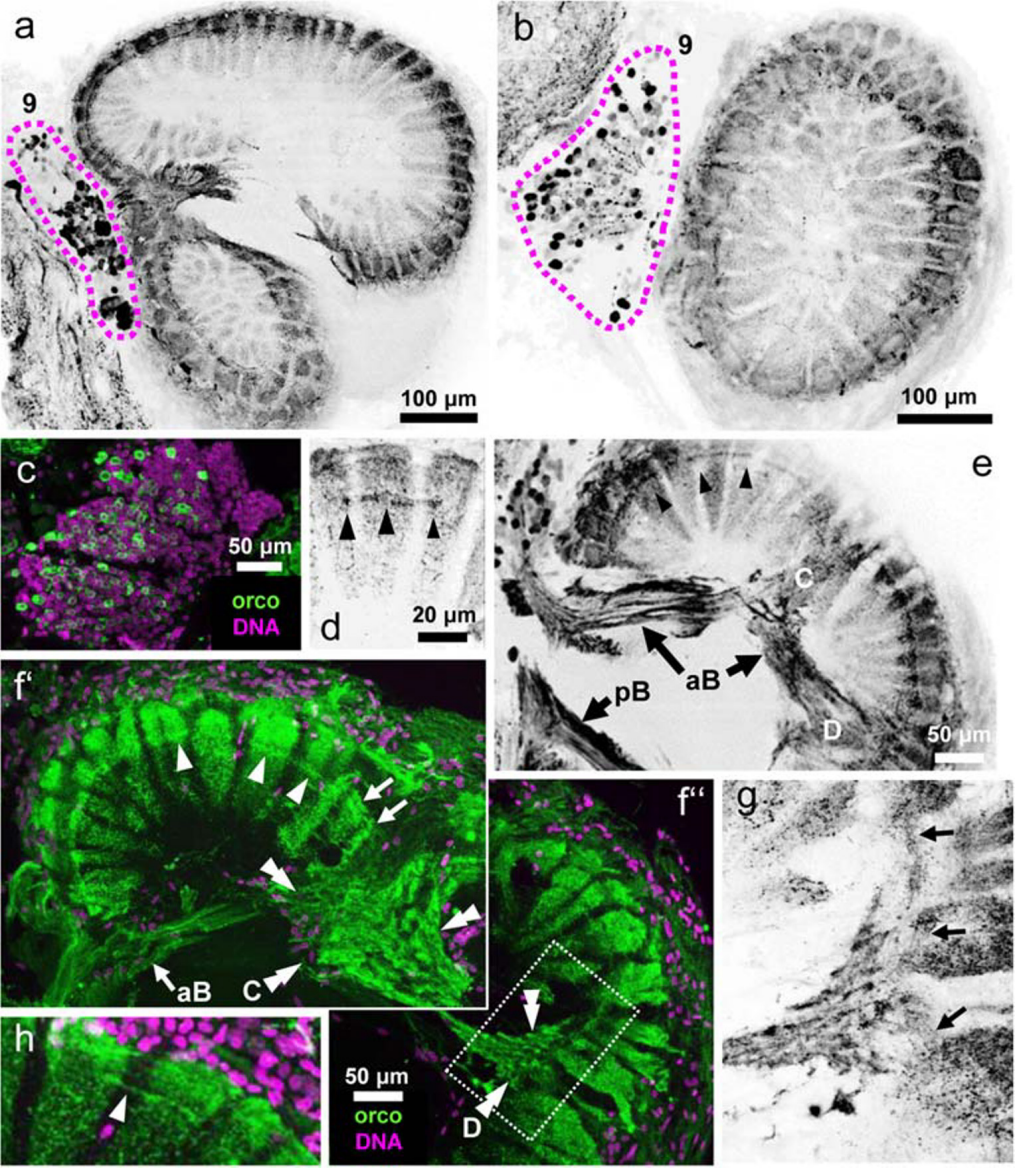
Orcokinin-immunoreactivity in local olfactory interneurons. **a**, **b** successive horizontal vibratome sections showing labeled somata in cell cluster (9) encircled by magenta line and strong immunoreactivity in the glomerular layer. **c** Within cluster (9), orcokinin-immunoreactive somata (green) are surrounded by other cells identified with a DNA marker (magenta). **d** Higher magnification of labeled glomeruli. Arrowheads point to a transverse band of strongly labeled material at the subcap level. **e** The anterior bundle (aB) of immunoreactive neurites projects into the lobe via the medial foramen (compare Fig. 2a) and extend towards non glomerular neuropils C and D. Arrowheads point to a transverse band of strongly labeled material at the subcap level. (f‘, f“)higher magnification of the anterior part of the lobe (montage; orcokinin immunoreactivity is shown in green, and nuclei in magenta) showing the anterior bundle projecting to non-glomerular neuropils C and D (double arrowheads). Note that no neurites from the anterior bundle directly innervate the glomeruli. Arrowheads point to a transverse band of strongly labeled material at the subcap level of the glomeruli. Arrows identify tangential fibers, which, from non-glomerular neuropil C, extend laterally towards the glomerular layer. The boxed area is magnified in (g). (g) Higher magnification of non-glomerular neuropil D. Arrows identify tangential fibers, which, from non-glomerular neuropil D, extend laterally towards the glomerular layer. (h) Higher magnification of the distal part of the glomerular layer. The arrowhead identifies interglomerular fibers connecting two glomeruli. aB anterior bundle, pB posterior bundle, letters C/D: non-glomerular neuropils C and D

All glomeruli in the lobe display strong immunoreactivity against orcokinin throughout their entire volume (Figs. 3 and 4), and this signal does not only stem from the afferences (see section above) but also from a second source. Within cell cluster (9), which houses the somata of local olfactory neurons, numerous somata display immunoreactivity to orcokinin as shown in two successive vibratome sections of a single brain (dotted lines in Fig. 4a, b). Co-labeling with a DNA markers shows that the immunolabeled somata are surrounded by numerous other somata with different, unknown transmitter phenotype (Fig. 4c). The neurites of the labeled neurons enter the primary chemosensory center via the medial foramen to form the anterior and posterior bundle (aB, pB; Figs. 2e and 4e). The fibers in the posterior bundle target the non-glomerular neuropil E (data not shown), whereas fibers in the anterior bundle innervate non-glomerular neuropils C and D (Fig. 4e, f‘). We did not find any evidence that immunolabeled fibers from the bundles directly target the olfactory glomeruli from their proximal side. Rather, higher magnifications suggested that from non-glomerular neuropils C and D (double arrowheads in photomontage Fig. 4f‘, f“) immunolabeled fibrous material extends laterally into the glomerular array (arrows in Fig. 4f‘, f“, and g). Inside the glomeruli, labeling is strongest in the distal quarter (Fig. 4d), presumably because the signal from the afferents overlaps here with that of the interneurons. Conspicuous transverse bands of particularly strongly labeled material are located at the interface of this distal quarter of the glomeruli and their proximal part (arrowheads in Fig. 4d, e, and f‘), a location that matches the subcap/base interface (compare Harzsch and Hansson 2008). In some preparations, immunolabeled neurites were visible, which link these transverse bands of neighboring glomeruli (arrowhead in Fig. 4h). This suggests that the orcokinin-immunoreactive neurons belong to the “rim”-type of local olfactory interneurons, which are for interglomerular connections by extending tangential fibers across the glomerular array, as opposed to “base”-type neurons, which target the glomerular base from a proximal direction (terminology according to Schachtner et al. 2005).

### Additional elements mediating lateral interglomerular connections

**Fig. 5:**
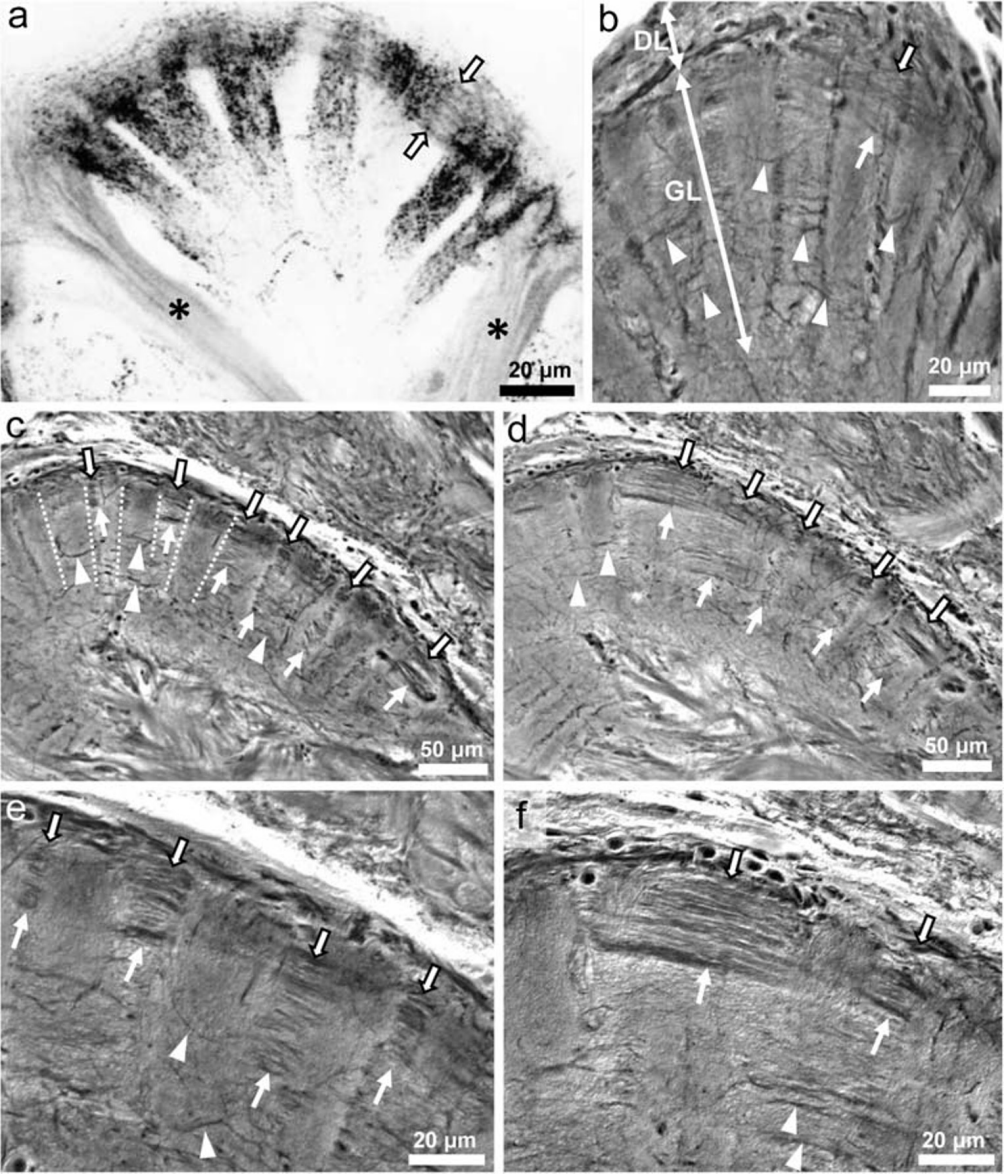
**a** Bundles of neurites with allatostatin-like immunoreactivity (asterisks) extend from the fibrous core of the lobe distally across the glomerular layer. There, the neurites change course to form a tangential fiber belt (paired arrows), which laterally connects multiple glomeruli at the subcap level. **b**–**f** silver impregnations showing the glomerular layer at different levels of the lobe in horizontal sections. Extensive belt-like neurite bundles (paired arrows) within the interglomerular space mediate lateral connections between the synaptic volumes of the glomeruli (outlined by dotted lines in C) and are concentrated in the upper quarter of the glomerular layer but are also located deeper. In addition, single, darkly stained horizontal fibers are visible in silver impregnated section (arrowheads). DL distal layer, GL glomerular layer

**Fig. 6:**
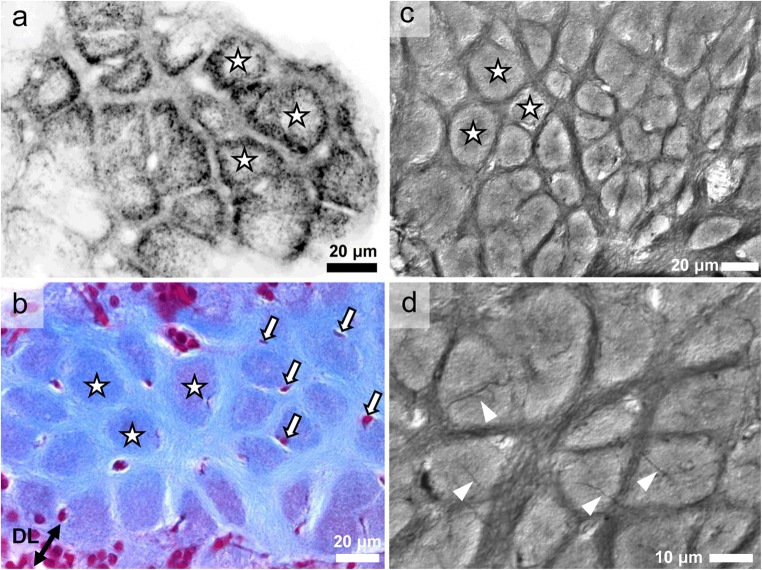
**a** Tangential sections of the glomeruli (asterisks) at the subcap level revealing allatostatin-like immunoreactive material in the interglomerular space. The glomeruli display strong immunoreactivity in an outer ring of the subcap area. **b** Azan-stained tangential section showing horizontal fibers in the interglomerular space between the synaptic areas of the glomeruli (asterisks). Arrows identify cross sections of finger-like protrusions of putative glial cells (compare Fig. 7a, b). **c**, **d** Silver-impregnated tangential sections at the subcap level reveal that the entire interglomerular space is densely filled with tangential fibers. Arrowheads **in d** identify single, darkly stained transglomerular horizontal fibers. DL distal layer

We had previously reported that *C. clypeatus* possesses local olfactory interneurons with allatostatin-like immunoreactivity (AST-ir) with somata in cell cluster (9), which also belong to the “rim”-type (Polanska et al. 2012). New preparations revealed bundles of neurites with AST-ir (asterisks in Fig. 5a), which, from the fibrous core of the lobe, extend distally across the glomerular layer. There, the neurites change course to form a tangential fiber belt (paired arrows in Fig. 5a), which laterally connects multiple glomeruli at the subcap level. Tangential sections of the lobe also revealed immunoreactive material in the interglomerular space (Fig. 6a). Consistent with our previous observations (Polanska et al. 2012), the glomeruli themselves (asterisks in Fig. 6a) showed strong AST-ir in an outer ring of the subcap area. Furthermore, silver impregnations revealed extensive tangential fiber bundles within the interglomerular space (paired arrows in Fig. 5b–f), which appear to connect the glomeruli laterally. These tangential neurite bundles are consistently visible in the upper quarter of the glomerular layer but also deeper. Silver-impregnated (Fig. 6c, d) and Azan-stained (Fig. 6b) tangential sections showed that in this area, the interglomerular space is densely filled with horizontal fibers. In addition to the belt-like neurite bundles, single, darkly stained horizontal fibers are visible in the center of the glomeruli in silver-impregnated sections (arrowheads in Figs. 5b–f and 6d).

### Blood vessels and glial cells within the glomerular layer

**Fig. 7:**
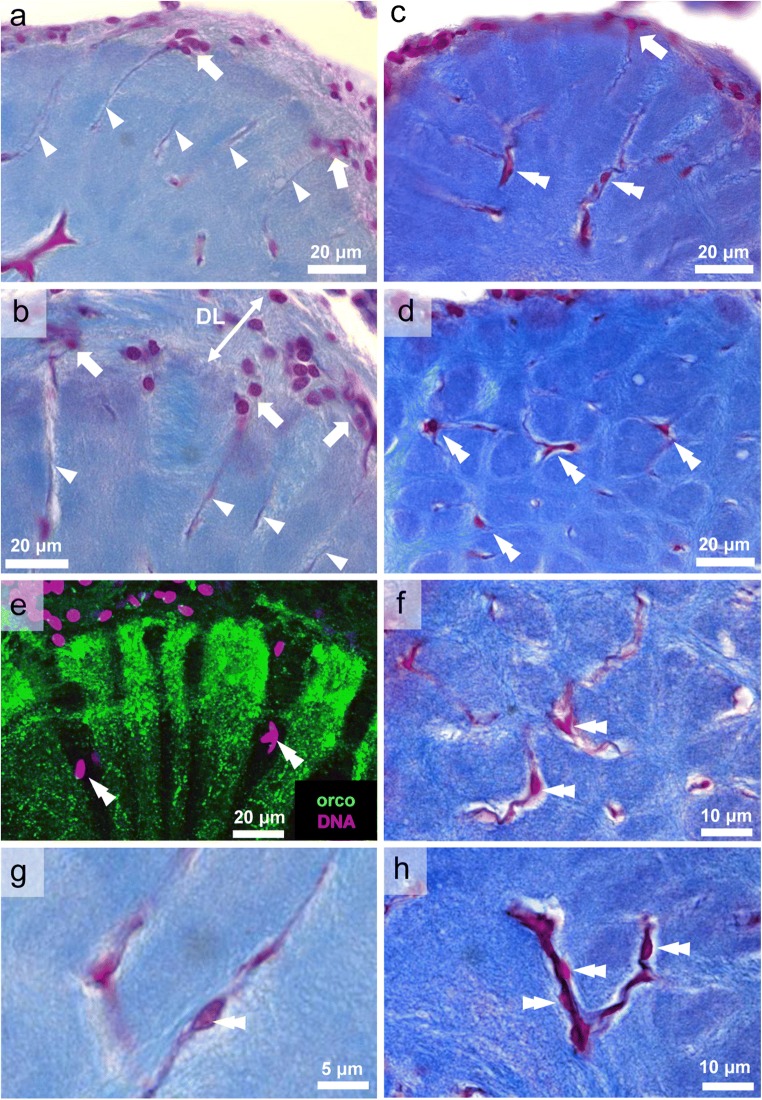
**a**, **b** Azan-stained sections of the glomerular layer show fine, elongate profiles between glomeruli (arrowheads), which in some cases emerge from clusters of cell nuclei (arrows; see also **c**) of putative glial cells within the distal layer of the lobe. **c**, **d** Azan-stained sections deeper within the glomerular layer, the profiles of branched blood vessels of the capillary network are visible (double arrowheads). **e** Labeling with a DNA marker (magenta) reveals individual cell nuclei (double arrowheads) within the orcokinin-ir glomerular layer. **f**–**h** Higher magnifications of capillaries to show associated cell nuclei (double arrowheads). DL distal layer

In Azan-stained sections that show the glomeruli in a longitudinal aspect, fine elongated profiles are visible, which are arranged in parallel to the glomeruli (arrowheads in Fig. 7a, b). These profiles were found to emerge from clusters of cell nuclei within the distal layer of the lobe (arrows in Fig. 7a, b) and their arrangement and morphology corresponds to a well characterized type of glia cells (Sandeman and Luff 1973; Orona et al. 1990; Helluy et al. 1996; Langworthy et al. 1997). Cross sections through the glomeruli at the subcap level also show the elongated profiles in cross section and reveal that almost every glomerulus seems to be associated with such a fine profile, which can be identified by its light red coloration (arrows Fig. 6b). Deeper within the glomerular layer, the profiles of branched blood vessels are visible (double arrowheads in Fig. 7c, d), which stand out because of their red color and which must belong to the capillary network of the lobe as previously described by Sandeman (1967) and Sandeman and Luff (1973). In higher magnifications of such capillaries, associated cell nuclei are visible (*double arrowheads* in Fig. 7f–h). Labeling with a DNA marker reveals only very few cell nuclei within the glomerular layer and these may be associated with blood vessels (Fig. 7e).

### The central projection-neuron hub

**Fig. 8:**
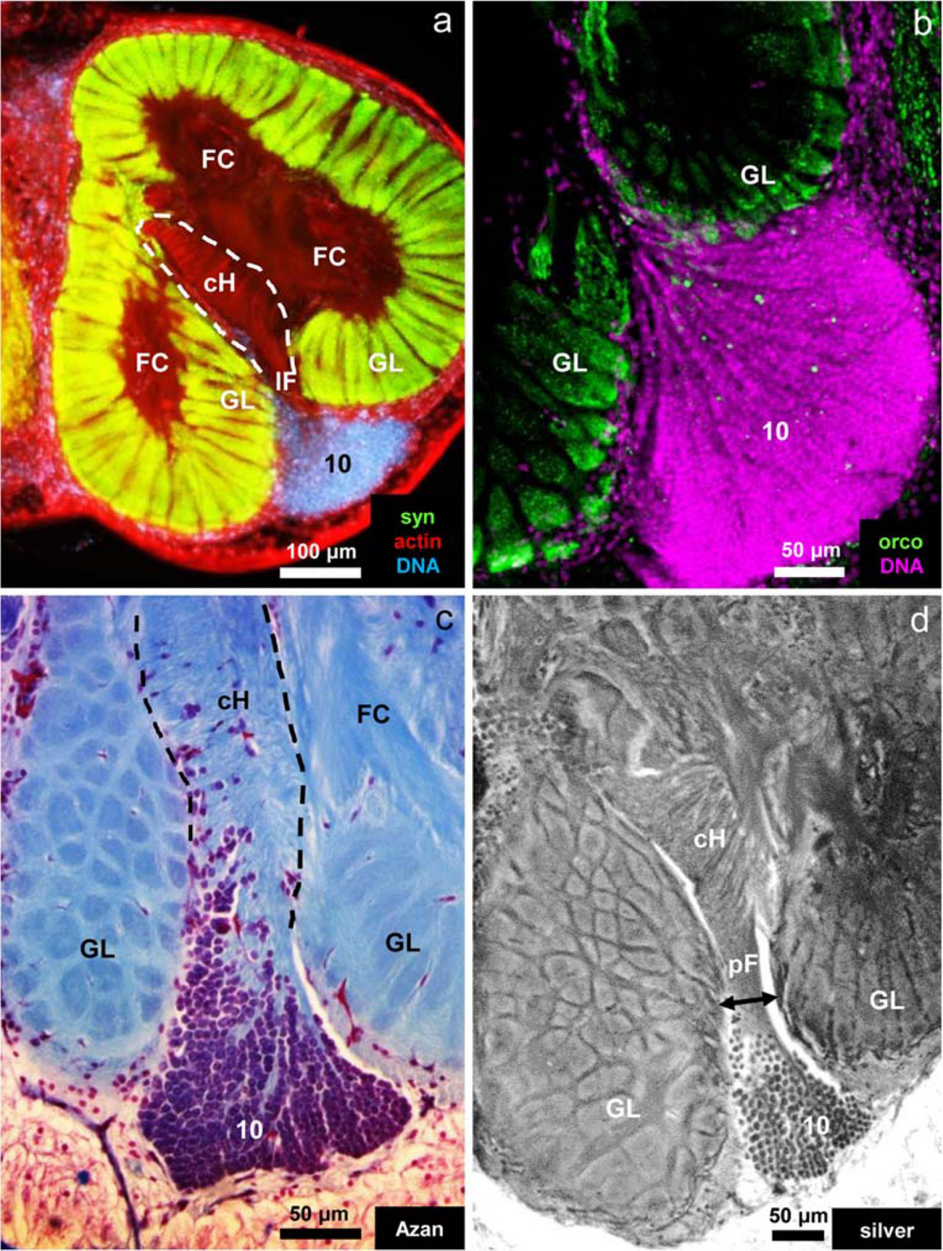
Neurites of projection neurons in cell cluster (10) project towards in the central projection neuron hub within the primary chemosensory center (the midline is to the left, and anterior to the top). **a** Triple labeling for synapsin-immunoreactivity (green), actin (red), cell nuclei (blue). **b** Orcokinin-immunoreactivity (green) and cell nuclei (magenta). **c** Azan-stain. **d** Silver impregnation. cH central projection neuron hub, FC fibrous core, GL glomerular layer, pF posterior foramen, number 10: cell cluster (10)

**Fig. 9:**
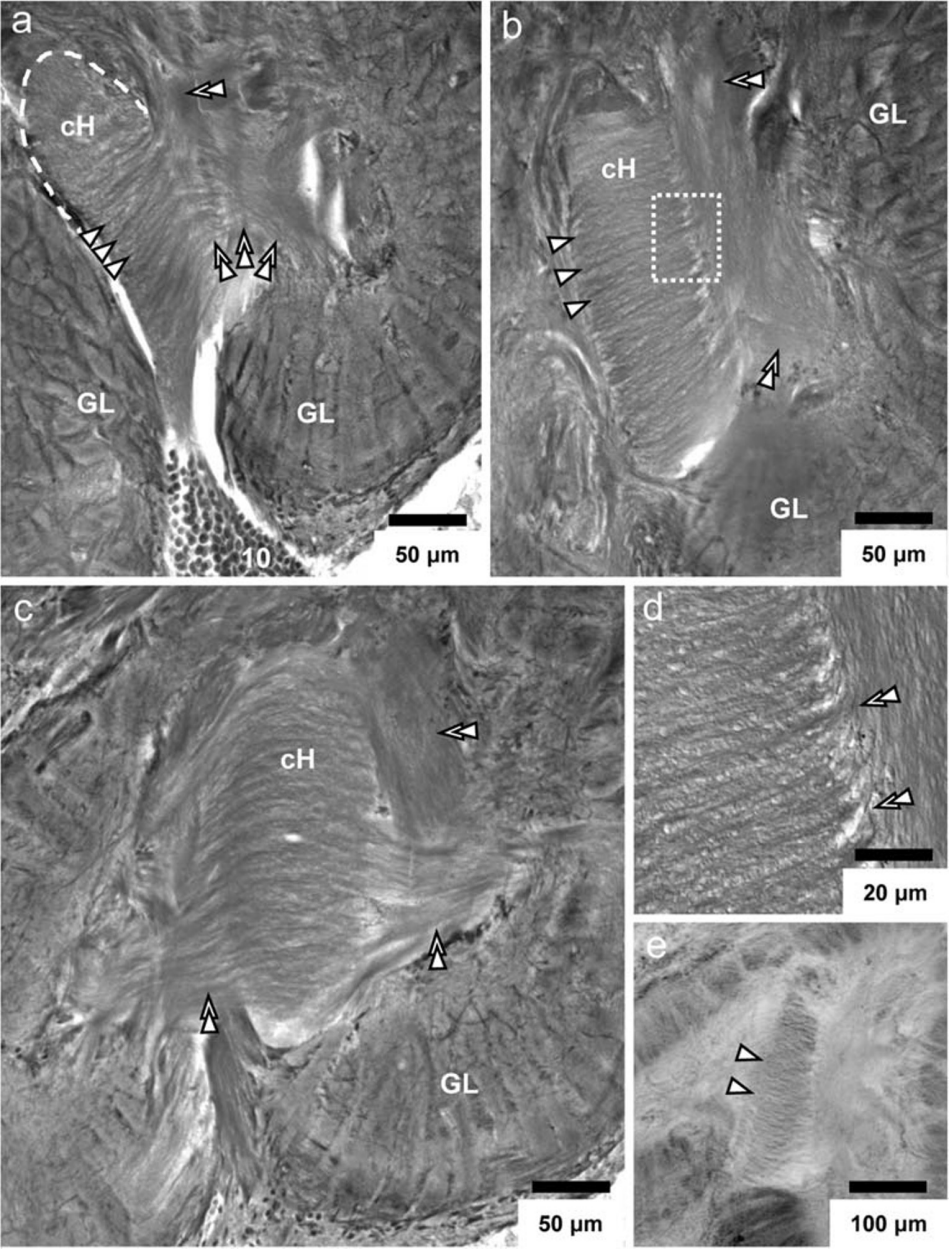
**a**–**d** Silver impregnations visualize the neurites within the central projection neuron hub. **a** Neurites emerging from cell cluster (10) enter the central hub where they get arranged in a parallel array (*arrowheads*). An array of parallel neurites (three *double arrowheads*) exits the central projection neuron hub (cH) and courses posterior-laterally to enter the proximal base of the glomerular layer. Another fiber tract exits the cH anteriorly (*double arrowhead*). **b** Higher magnification of the central projection neuron hub to show the parallel arrangement of neurites (*arrowheads*). Double arrowheads identify fiber bundles, which exit the hub. The boxed area is shown in D at a higher magnification. **c** In a different section plane, another field of parallel neurites becomes visible, which exits the central hub in a medial direction (*double arrowheads* to the left). **d** Higher magnification of the boxed area in **b**. Note that the parallel neurites take a sharp bend anteriorly (double arrowheads) as they exit the hub. **e** The parallel arrangement of neurites (arrowheads) within the hub as visualized by a marker for actin (black-white inverted fluorescent image). cH central projection neuron hub, GL glomerular layer, number 10: cell cluster (10)

In decapod crustaceans, cell cluster (10) houses the somata of olfactory projection neurons (Sandeman et al. 1992), which extend neurites into the primary chemosensory center and have axons, which assemble in the projection neuron tract to target the lateral protocerebrum (summarized in Schachtner et al. 2005, Harzsch and Krieger 2018). In *C. clypeatus,* DNA markers (Fig. 8a, b) and classical histology (Fig. 8c, d) reveal that cluster (10) is composed of thousands of somata of nearly identical size. In some preparations, the somata appear to be arranged in lines pointing towards the posterior foramen (Fig. 8b, c). Silver impregnations show that bundles of parallel neurites emerge from this cluster to enter the lobe via the posterior foramen to target the central projection neuron hub (Figs. 8d and 9a). Within the hub, the axons get sorted in a way that they are arranged in parallel to each other (arrowheads in Fig. 8a, b). This texture of strictly aligned fibers within the hub is also visible in specimens labeled with a probe against actin (Fig. 8e). In different section planes, different arrays of fibers can be seen to emerge from the central projection neuron hub to extend towards the proximal side of the glomerular layer: (i) one array of fibers that extends in posterior lateral direction (double arrow heads in Fig. 9a), (ii) another array that extends laterally (Fig. 9b, c), and (iii) a further array that extends medially (Fig. 9c). In some section planes (Fig. 9d), the fibers that exit the hub can be seen to take a sharp bend anteriorly and to assemble in a tract towards the periphery of the lobe (double arrowheads in Fig. 9a, b).

## Discussion

### Afferences

**Fig. 10:**
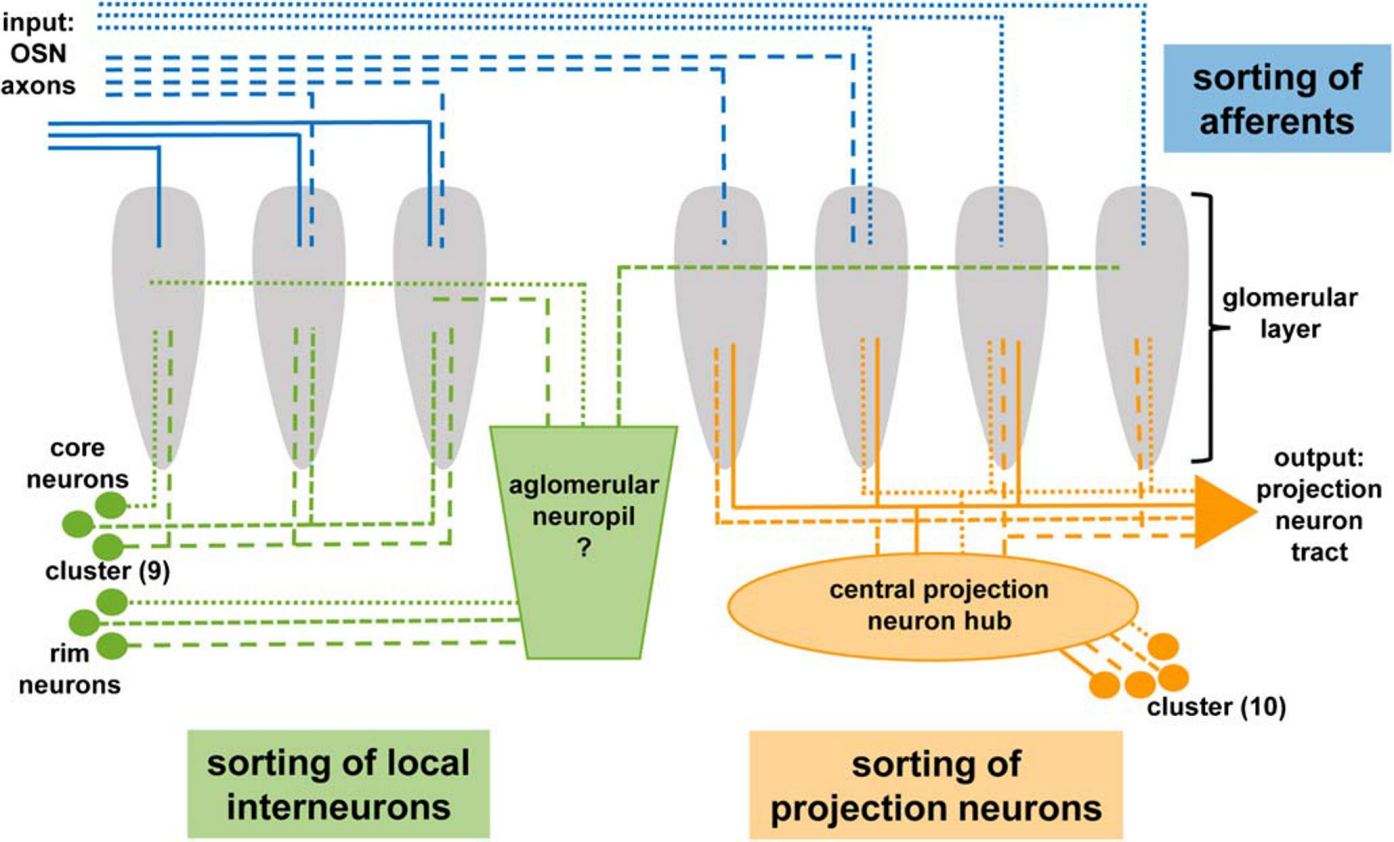
Model summarizing the innervation of the olfactory glomeruli in *C. clypeatus* with afferents, local olfactory interneurons and olfactory projection neurons. The colored boxes indicate presumable sites where neurites are sorted towards their target structures. For further explanation, see text

The deutocerebral antennal pair of malacostracan crustaceans bears at least two classes of sensilla, specialized, unimodal olfactory sensilla, the aesthetascs, and bimodal, i.e.chemo- and mechanosensory sensilla (review Hallberg and Skog 2011; Schmidt and Mellon 2011; Derby et al. 2016). As the afferent nerve from this appendage enters the brain, the axons progress across a large axon-sorting zone in which the input from the two classes of sensilla is segregated to target separate sensory neuropils (Schmidt and Ache 1992; Schmidt et al. 1992). Here, we will restrict our discussion to the aesthetasc input into its primary olfactory center. In crayfish and spiny lobsters, the axons of the olfactory sensory neurons form a plexus-like distal layer around the periphery of the lobe from which fascicles segregate, and the afferents enter the glomeruli vertically (Fig. 10; Sandeman and Luff 1973, Blaustein et al. 1988, Mellon and Alones 1993, Schmidt and Ache 1992, Sandeman and Sandeman 2003). The afferences from a cluster of olfactory sensory neurons associated with one aesthetasc sensillum in crayfish were shown to target many glomeruli of the lobe (Mellon and Munger 1990; Sandeman and Denburg 1976) so that their projection pattern appears to be non-topographic (review Mellon and Alones 1993). Every aesthetasc sensillum is associated with an identical set of different types of olfactory sensory neurons that are characterized by different odorant receptor proteins (Mellon 2007). This suggests that each cluster of olfactory sensory neurons has an identical composition, regardless of its position along the antennae. Beltz et al. (2003) proposed that “an animal’s entire chemoreceptive range is represented by the olfactory sensory neurons associated with each individual aesthetasc sensillum.” In spiny lobsters, the cap region of the glomeruli represents the major input region where the afferent axons branch densely (Schmidt and Ache 1992). Nevertheless, some axons also project down the entire length of the glomeruli into the subcap and base regions in this species. In spiny lobsters (Schmidt and Ache 1992) and terrestrial hermit crabs (Tuchina et al. 2015), evidence obtained from antennal backfills and silver impregnation techniques indicates that both uniglomerular and multiglomerular terminations of the afferents occur frequently (Fig. 10).

Acetylcholine likely represents the main transmitter of the olfactory sensory neurons (review Schachtner et al. 2005). This study for the first time reports that the afferents also contain orcokinin-like neuropeptides, although its function in olfactory transmission remains unknown. In the lobster *Homarus americanus,* identified Ala^13^-orcokinin has been shown to modulate pyloric rhythms especially of the well-known so-called lateral pyloric (LP) and the pyloric (PY) neurons seemingly influenced by the peptide released from the multitransmitter pyloric suppressor neurons (containing also histamine and FLRFamides) located in the inferior ventricular nerve. This nerve connects the brain with the stomatogastric nervous system in several decapods (Li et al. 2002).

The distal layer of the lobe with its plexus-like texture must function as an axon sorting zone in which the axons get distributed to their target glomeruli (Fig. 10). Contrary to insect olfactory systems in which glial cells play important roles in axonal sorting during ontogeny (reviews e.g. Oland and Tolbert 1996, 2003, 2011), we know very little in crustaceans about the rules governing the developmental segregation of afferent axons to the glomerulus (review Harzsch and Krieger 2018). Our study showed the presence of numerous cell nuclei in the distal layer in addition to the presence of the plexus formed by OSN-afferences. Morphological characteristics of the distal layer were already described in the crayfish *Cherax destructor* (Sandeman and Luff 1973) and subsequently were also reported for the crayfish *Procambarus clarkii* and the spiny lobster *Panulirus argus* (Blaustein et al. 1988). Sandeman and Luff (1973) described that glial cells in the distal layer send finger-like projections deep into the lobe in parallel to the glomeruli and are located in the interglomerular space. The authors’ description matches the situation we found in Azan-stained sections. Cells with a similar location and morphology were also described to display histamine immunoreactivity in spiny and clawed lobsters (Orona et al. 1990; Langworthy et al. 1997) and to define the glomerular borders (Helluy et al. 1996). Future studies using glial cell markers such as antisera against glutamine synthetase (Linser et al. 1997; Harzsch and Hansson 2008) may reveal the arrangement and morphology of the glial cells in the distal layer in more detail. In insects, various classes of glia cells play important roles during the development of the primary olfactory center, e.g., in that they organize ingrowing afferents in an axon-sorting zone, interact with ingrowing afferents to initiate the formation of protoglomeruli, and stabilize the emerging glomeruli during ontogenesis (reviews Oland and Tolbert 1996, 2003, 2011). Such aspects are largely unexplored in crustaceans (Helluy et al. 1993, 1996) so that thoroughly analyzing the ontogeny of their olfactory glomeruli, specifically possible interactions of entering afferents, the developing local interneurons and glial cells may be a warranting project.

### Local interneurons: elements mediating lateral interactions between glomeruli

Within the glomerular layer, the afferents synapse with local olfactory interneurons and the projection neurons whose axons target the lateral protocerebrum (Fig. 10; reviews Sandeman and Mellon 2002, Schachtner et al. 2005, Schmidt 2007, Schmidt and Mellon 2011, Derby and Weissburg 2014, Sandeman et al. 2014). Two morphological classes of the local interneurons were reported (Fig. 10), a first class that primarily connects to the cap and subcap regions of the glomeruli (“rim” interneurons) and a second class that primarily branches within the base of the glomeruli, invading them from their proximal side (“core” interneurons; Schmidt and Ache 1996, Wachowiak et al. 1997; review Schachtner et al. 2005). Other local interneurons show different morphologies (Schmidt and Ache 1996; Wachowiak et al. 1997), which do not fall within these two classes (review Schachtner et al. 2005).

Local olfactory interneurons also display a large diversity in their neurochemistry. In an extensive review, Schachtner et al. (2005) listed different neuroactive substances that were localized in crustacean local olfactory interneurons: gamma-aminobutyric acid (GABA), nitric oxide, the biogenic amines serotonin, histamine, and dopamine, and the neuropeptides small cardioactive peptide (SCPb), substance P/tachykinin, proctolin, FMRFamide-related peptides (FaRPs), and enkephalin-like peptides. Yasuda-Kamatani and Yasuda (2006) added allatostatin (AST)-like peptides, FaRPs, orcokinin, tachykinin-related peptides, and SIFamide to this list. “Core” interneurons were shown to possess neuroactive substances as diverse as nitric oxide, serotonin, histamine, and FaRPs (review Schachtner et al. 2005), whereas “rim” interneurons contain GABA (Schachtner et al. 2005), FaRPs, and ASTs (Polanska et al. 2012). The present reports add orcokinin-immunoreactive neurons to the list of “rim” interneurons. The question how these diverse types of local olfactory interneurons during development find their individual set of target glomeruli and the correct glomerular compartment to innervate remains largely unexplored. Because interneurons with FaRP immunoreactivity (Harzsch and Hansson 2008), AST-immunoreactivity (Polanska et al. 2012), and orcokinin-immunoreactivity (present report) target the glomerular array via the non-glomerular neuropils, we propose that for the “rim” type of local interneurons in *C. clypeatus* the non-glomerular neuropil may act as a kind of interface to guide the axons towards their tangential course across the glomerular array (Fig. 10).

Many of the individually identified local interneurons branch in large ensembles of glomeruli, in some cases up to 85% of the entire glomerular layer (Wachowiak et al. 1997). Certain types of “rim” interneurons primarily invade the subcap region and most likely provide distinct lateral connections across the glomerular array (Schmidt and Ache 1996). These connections were termed “interglomerular fibers” in crayfish (Blaustein et al. 1988). Because some of them contain GABA, they may modulate the input of the olfactory sensory neuron to the projection neurons in spiny lobsters (Wachowiak et al. 1997). These rim neurons may mediate lateral inhibitory interactions through presynaptic inhibition of afferents (Schmidt and Ache 1996a).

Using classical histology, the current report presents additional evidence for elaborate lateral connections across the glomerular array. In insects, a comparison and subsequent modulation of global activity across all sensory neurons was suggested to represent one essential step for transforming the input of olfactory sensory neuron into an odor-specific combinatorial code of glomerular activity (Martin et al. 2011; Wilson 2013; Galizia 2014; Szyszka and Galizia 2015). In insect olfactory systems, a network of lateral inhibitory local interneurons the neurites of which extend across the glomerular may function as a gain control in which activity surrounding specific glomeruli is suppressed at high-odor concentrations and the interglomerular contrast is enhanced (Galizia 2014; Szyszka and Galizia 2015). The glomeruli of malacostracan crustaceans are arranged in a strictly radial array with each glomerulus parallel to its immediate neighbors. The subcap connections by interglomerular fibers were proposed to be the essential driver forcing the glomeruli to display this same configuration in all malacostracans (Harzsch and Krieger 2018). The parallel arrangement and the regular spacing of the glomeruli has the effect that the subcap compartment of a given glomerulus is similarly close to all of its immediate neighbors and this “constructional feature may represent an optimal layout in the light of wiring economy and synchronizing activity with regard to the functioning of lateral inhibition in crustaceans” (Harzsch and Krieger 2018). Considering that with increased input, the inhibitory interactions between the glomeruli are essential (Martin et al. 2011) in crustaceans with about one thousand glomeruli such as *C. clypeatus* (Tuchina et al. 2014), lateral inhibitory interactions mediated by the “rim” type of local interneurons may be crucial for controlling the activity within the network. Future work will have to reveal the significance and possible actions and roles of the diverse neuropeptides occurring in local interneurons; here they most likely act as neuromodulators fine-tuning the circuitries within the olfactory glomeruli (Schachtner et al. 2005).

### The central projection neuron hub

The projection neurons constitute the output pathway of the malacostracan olfactory system. They typically have small somata (diameter 4.5 μm; Wachowiak and Ache 1994) and also their axons are extremely thin (200 nm in crayfish; Mellon et al. 1992). These neurons are located within cluster (10) and that their dendrites innervate the principal glomerular output regions, the base (Figs. 10; Mellon et al. 1992, Mellon and Alones 1993, Wachowiak and Ache 1994, Schmidt and Ache 1996). The axons provide input to the bilaterally paired medulla terminalis/hemiellipsoid body complex, a higher order, multimodal processing area in the protocerebrum (reviews Sandeman et al. 2014, Harzsch and Krieger 2018). The axons form a large tract, the projection neuron tract, which represents a conspicuous anatomical landmark in malacostracan brains (Fig. 1). Collateral axons within this tract to the brain’s contralateral side form a characteristic chiasm in the center of the brain (Fig. 1; Sandeman et al. 1995; Schmidt and Ache 1996). Projection neurons represent the least understood population of neuronal elements in the crustacean olfactory system, and we do not have any information at all if the findings described above for higher malacostracans hold true for other crustaceans as well. Because their neurochemistry is unclear, studies have so far mostly relied on mass fills or single-cell tracer injections (review Schachtner et al. 2005). Although Polanska et al. (2012) showed that, in crayfish, projection neurons can be labeled with an antiserum against the neuropeptide SIFamide (perhaps acting as a co-modulator for a yet unidentified neurotransmitter), this is not the case in the giant robber crab *Birgus latro*, another member of the Coenobitidae (Krieger et al. 2010). In *C. clypeatus*, the central projection neuron hub has until now escaped our notice (Harzsch and Hansson 2008; Polanska et al. 2012; Tuchina et al. 2015), and, to our knowledge, this structure has not yet been described in any other malacostracan crustacean. This fact highlights the merits of using classical neuroanatomical methods in conjunction with immunohistochemistry. We assume that projection neurons in *C. clypeatus* display an innervation pattern of similar complexity as that described for the spiny lobster (Wachowiak and Ache 1994). Therefore, a more detailed analysis of the functional logic in this axon sorting zone with additional methods is a warranting enterprise to gain deeper insights into the rules that govern the projection neuron readout of the cholinergic and orcokininergic OSN-activities across the glomerular array in this system.
